# Alkoholprävention am Arbeitsplatz: Aktuelle Konzepte zur betrieblichen Suchtprävention und Suchthilfe

**DOI:** 10.1007/s00103-021-03337-6

**Published:** 2021-05-22

**Authors:** Elisabeth Wienemann, Anja Wartmann

**Affiliations:** 1 Institut für interdisziplinäre Arbeitswissenschaft, Leibniz Universität Hannover, Stolzestr. 41, 30171 Hannover, Deutschland; 2 Gesundheitsamt Braunschweig, Braunschweig, Deutschland

**Keywords:** Alkoholkonsum, Arbeitssicherheit, Gesundheitsförderung, Suchtberatung, Gesundheitsgefährdung, Alcohol consumption, Safety at work, Health promotion, Addiction counseling, Health hazards

## Abstract

Zur Vorbeugung von Alkoholproblemen am Arbeitsplatz und zur Hilfe bei Suchtgefährdung hat sich das Standardmodell der betrieblichen Suchtprävention und Suchthilfe bewährt. Kernelemente sind: 1) Information und Aufklärung, 2) frühzeitige Interventionen, 3) Qualifizierung und Beratung von Personalverantwortlichen und 4) Hilfeangebote für Beschäftigte. In diesem Beitrag wird zunächst die historische Entwicklung der betrieblichen Alkohol- und Suchtprävention kurz beschrieben, Forschungsergebnisse werden dargestellt und danach wird auf Maßnahmen zur Prävention, zur Intervention bei Suchtgefährdung und zur Reduzierung des Alkoholkonsums am Arbeitsplatz eingegangen.

In den 1970er-Jahren wurden betriebliche Suchtpräventionsprogramme nach US-amerikanischem Vorbild in Deutschland etabliert. Im Jahr 2006 veröffentlichte die Deutsche Hauptstelle für Suchtfragen (DHS) erstmals die „Qualitätsstandards in der betrieblichen Suchtprävention und Suchthilfe“ und ermöglichte allen Betrieben den Zugriff auf fachlich abgestimmte und rechtlich aktuelle Materialien. Das ist bedeutsam, um angemessene Maßnahmen zur Regulierung und Reduzierung des Alkoholkonsums am Arbeitsplatz zu treffen. Zum Präventionsauftrag der Führungskräfte gehört die Unterweisung der Beschäftigten zur Vermeidung gesundheitlicher Gefährdungen. Sobald Verstöße gegen die Arbeitssicherheit vorliegen, sind Vorgesetzte zudem verpflichtet einzugreifen. Generell sollten sie bei Auffälligkeiten möglichst frühzeitig intervenieren. Bei riskantem Suchtmittelgebrauch und Suchtgefährdung wird nach einer gestuften Gesprächsfolge verfahren. Unterstützungsangebote von internen oder externen Beratungsstellen werden empfohlen.

Alkohol- und Suchtprävention ist Teil des betrieblichen Gesundheitsmanagements. Besonders nachhaltig wirkt sie dort, wo das Programm in einer Betriebs- oder Dienstvereinbarung festgeschrieben wurde und verbindlich umgesetzt wird.

## Abstract (en)

The standard workplace addiction prevention and addiction counselling programmes for preventing alcohol problems in the workplace and helping those at risk of addiction have been proven to be successful. The key elements are, firstly, information and education; secondly, early intervention if conspicuous behaviour in the workplace is identified; thirdly, qualification and counselling by human resources staff; and, finally, assistance for employees with alcohol or other substance addiction problems.

In the 1970s, corporate addiction prevention programs based on the American model were established in Germany. In 2006 the German Centre for Addiction Issues (DHS) published for the first time the “Guidelines on Addiction Prevention and Assistance Programs in Companies” as a quality standard. Since then, all companies or public administrations have had open access to specialised knowledge and current legal materials. This is important in order to regulate and reduce alcohol consumption in the workplace. The prevention mandate of managers includes instructing employees on how to avoid health hazards. As soon as there are occupational safety violations, superiors are obliged to intervene. In general, they should intervene as early as possible in the case of irregularities caused by alcohol consumption. In the case of high-risk drug use and risk of addiction, the procedure is based on a tiered sequence of interviews. Support offers from internal or external advice centres are recommended.

Alcohol and addiction prevention is part of occupational health management. It has a particularly lasting effect where the program has been written in a company or service agreement and is implemented in a binding manner.

## Einleitung

Die Alkohol- und Suchtprävention ist ein bedeutendes Handlungsfeld im betrieblichen Personal- und Gesundheitsmanagement. Information und Aufklärung der Beschäftigten sowie frühzeitige Interventionen bei Auffälligkeiten am Arbeitsplatz tragen zum Erhalt und zur Wiederherstellung ihrer physischen und psychischen Gesundheit bei. Da sich auf diese Weise dramatische Verläufe bei Suchtproblemen genauso wie lange krankheitsbedingte Abwesenheiten vermeiden lassen, profitieren Beschäftigte und Betriebe gleichermaßen davon.

In diesem Übersichtsartikel werden zunächst die Entwicklung und der aktuelle Stand betrieblicher Suchtpräventionskonzepte aufgezeigt. Ziel ist es, ihre Wirkung, Verbreitung und Relevanz anhand der vorliegenden Datenbasis zu belegen. Betriebliche Suchtprävention, das kann verdeutlicht werden, ist nicht allein auf der Verhaltensebene und den Problemen einzelner Beschäftigter zu denken. Sie zielt in hohem Maße auch auf die Verhältnisse, wie z. B. suchtfördernde Arbeitsbedingungen, und die Prävention von Gefährdungen. Zudem nimmt sie die strukturelle Verankerung des betrieblichen Suchtpräventionsprogramms in den Blick. Die *Qualitätsstandards für die betriebliche Suchtprävention und Suchthilfe* der Deutschen Hauptstelle für Suchtfragen (DHS; [1]) liefern ein fachlich und rechtlich abgestimmtes Konzept für die Umsetzung in der Praxis und unterstreichen seine Relevanz im Sinne der *gesicherten arbeitswissenschaftlichen Erkenntnisse* [2]. Forschungen zur erwiesenen Wirksamkeit im Sinne der *evidenzbasierten Medizin *[3] fehlen allerdings bisher.

Im darauffolgenden Kapitel wird dargelegt, inwiefern der betriebliche Kontext ein wichtiges Setting bietet, um zum einen erwachsene Menschen im Berufsalter regelmäßig auf die Wirkung und die Funktion von Alkohol aufmerksam zu machen und zum anderen Regelungen zur Konsumreduzierung zu vereinbaren. Die Datenlage, die sich mit dem riskanten Alkoholkonsum befasst, spricht dafür, diese Möglichkeiten systematisch zu nutzen [4].

Im Anschluss werden die Verfahren vorgestellt, die als wichtige Instrumente des Personal- und Gesundheitsmanagements die rechtlich und fachlich angemessenen Interventionen bei riskantem Konsum und Suchtgefährdung erleichtern. Eine wichtige Rolle nehmen dabei die Führungskräfte mit ihren Fürsorge- und Präventionspflichten ein. Von ihrer Qualifikation und Handlungsbereitschaft hängt wesentlich der Erfolg der Interventionen ab [5].

Verbindliche Angebote der betrieblichen Suchtprävention zur Information und Aufklärung sowie zur Beratung und Unterstützung werden in einem weiteren Kapitel beschrieben. Sie tragen dazu bei, die dafür erforderlichen strukturellen Voraussetzungen mitzugestalten. Die dafür erforderlichen strukturellen Voraussetzungen werden in einer Vereinbarung festgelegt [1].

Abschließend werden Maßnahmen zur übergreifenden Prävention angesprochen. Sie gewinnen angesichts sich verändernder Anforderungsstrukturen im Betrieb an Bedeutung [6]. Im Rahmen des Gesundheitsmanagements ist die betriebliche Suchtprävention dafür ein wichtiger Kooperationspartner.

Für die Bearbeitung des Themas wird im Beitrag im Wesentlichen auf Literatur zurückgegriffen, in die Ergebnisse grundlegender Literaturrecherchen eingeflossen sind, v. a. die Monografie *Vom Alkoholverbot zum Gesundheitsmanagement – Entwicklung der betrieblichen Suchtprävention von 1800 bis 2000* [7], für die in den 1990er-Jahren Literaturdatenbanken herangezogen wurden wie PsycLIT, Psyndex, APA PsycInfo sowie die Bestände der Staatsbibliothek Hamburg, der Deutschen Bibliothek (Frankfurt am Main bis 1990), der Amerika-Gedenkbibliothek, des Archivs für Soziale Fragen (beide in Berlin), der Niedersächsischen Landesbibliothek und des Archivs der Deutschen Hauptstelle für Suchtfragen (DHS). Der Rechercheprozess wurde für neuere Veröffentlichungen unter Einbeziehung von PsychData, PubMed und der Gesundheitsberichterstattung des Bundes jeweils auf den neuesten Stand gebracht.

## Rahmenbedingungen und Konzepte zur betrieblichen Alkohol- und Suchtprävention

### Entwicklung und Einordnung der betrieblichen Suchtpräventionsprogramme

Suchtprävention und Suchthilfe^1^ im Betrieb stehen in Deutschland in einer langen Tradition. Schon Ende des 19. Jh. gab es intensive Bemühungen, den Branntweinkonsum am Arbeitsplatz zu regulieren und einzuschränken. Die Mäßigkeitsbewegung legte Flugschriften zur Verteilung an Beschäftigte aus und etablierte Ansprechpersonen im Betrieb [7].

Anfang der 1940er-Jahre wurde am Yale Center of Alcohol Studies in den USA *das medizinische Modell des Alkoholismus *beschrieben, das der weltweiten* Anerkennung der Alkoholabhängigkeit als Krankheit* zugrunde liegt. 1948 wurde das in Yale mit Expertise aus der Betriebsmedizin und der Selbsthilfe entwickelte *Occupational Alcoholism Program *vorgestellt. Mit staatlicher Unterstützung konnte das Modell in den folgenden Jahrzehnten in den USA in öffentlichen und privaten Betrieben eingeführt werden. Als Employee Assistance Program (EAP) wurde es danach weltweit bekannt [7, 8].

Kernelement des Programms ist ein *betrieblicher Prozess, der entlang eines Stufenplans zu lösungsorientierten Interventionen führt *mit dem Ziel, durch Alkohol- bzw. Suchtmittelgebrauch auffällig gewordene Beschäftigte konstruktiv zu unterstützen. In den Stufengesprächen werden betroffene Personen zunächst von ihren Vorgesetzten dazu motiviert, das beanstandete Verhalten zu verändern. Wenn erforderlich werden sie angehalten, intern oder extern fachliche Beratung und ggf. Therapie in Anspruch zu nehmen. Sofern dies aus eigener Kraft nicht (mehr) gelingt und die verminderte Kontrolle über den Konsum zu riskantem und schließlich gefährdendem Verhalten sowie zur Vernachlässigung arbeitsvertraglicher oder dienstlicher Pflichten führt, wird das nächste Stufengespräch im Stufenplan angesetzt. Erst bei fortgesetzt negativer Entwicklung und erneuten Auffälligkeiten im Arbeitsverhalten finden Gespräche in einem erweiterten Gesprächskreis statt und folgen disziplinarische Konsequenzen [1, 9].

Da die Suchtprävention und Suchthilfe viele Rechtsbereiche berühren, wird den Betriebsparteien empfohlen, eine schriftliche Betriebs- oder Dienstvereinbarung abzuschließen. Diese enthält u. a. eine transparente Beschreibung der Interventionsverfahren. Zudem regelt sie verbindlich die Qualifizierung der Personalverantwortlichen, die Informations- und Unterstützungsangebote für Beschäftigte sowie den Einsatz einer Beratung für Suchtfragen im Betrieb. Schließlich sichert sie auch den Persönlichkeits- und Datenschutz [1]. Alle hier aufgezählten Elemente gehören zu den einschlägigen Erfolgsfaktoren eines Alkohol- und Suchtpräventionsprogramms [5, 10].

Als das EAP-Konzept Anfang der 1970er-Jahre in der Bundesrepublik bekannt wurde, war „süchtiges Trinken als besondere Form des Alkoholismus“ 1968 gerade erst vom Bundessozialgericht als behandlungsbedürftige Krankheit anerkannt worden. Schon 1975 wurde der erste Stufenplan in einer Betriebsvereinbarung festgeschrieben. Statt der ursprünglich vorgesehenen frühen Intervention *bei Alkoholproblemen* wurde allerdings in den hiesigen Konzepten häufig erst ein Eingreifen bei *Symptomen der Alkoholkrankheit* empfohlen [4, 7]. Ohne eindeutige Diagnose schränkte das die Handlungssicherheit der Personalverantwortlichen erheblich ein.

Aufwind bekam die betriebliche Alkohol- und Suchtprävention in den 1990er-Jahren. Im Zuge des technisch-organisatorischen Wandels wurden die gesundheitlichen Folgen der steigenden mentalen und psychischen Anforderungen in den Betrieben sichtbar [11, 12]. Das 1996 eingeführte *Arbeitsschutzgesetz* (ArbSchG) formulierte zudem neue Präventionspflichten. Danach sind Arbeitende vor Gefahren für die physische und psychische Gesundheit möglichst zu schützen und Gefährdungen bei der Arbeit zu ermitteln und zu beseitigen [13]. Die betriebliche Suchtprävention liefert dazu aktive Beiträge und erprobte Instrumente.

**Figure 1:**
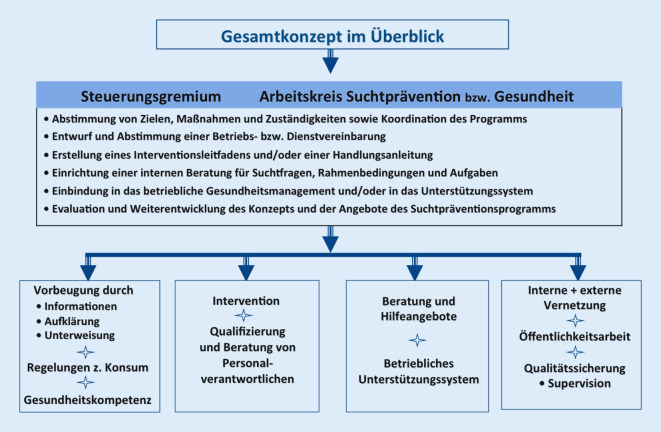

Im Jahr 2006 veröffentlichte die *Deutsche Hauptstelle für Suchtfragen (DHS) *auf der Basis einer Expertise [10] erstmals die *Qualitätsstandards für die betriebliche Suchtprävention und Suchthilfe*. Diese ermöglichen allen Betrieben jederzeit auf das Modell eines fachlich abgestimmten und rechtlich aktuellen Suchtpräventionsprogramms sowie auf viele begleitende Materialien zuzugreifen (Abb. 1; [1]).

### Wirkungen der betrieblichen Alkohol- und Suchtpräventionsprogramme

Die vielfältigen positiven Effekte der betrieblichen Alkohol- und Suchtprävention hierzulande lassen sich leider nicht umfassend belegen. Eine systematische Wirksamkeitsforschung für dieses Setting gibt es nicht, da für die betrieblichen Programme nur geringe Forschungsmittel zur Verfügung stehen. Selten kann auf repräsentative Studien zurückgegriffen werden [10, 14, 15]. Limitierend ist also festzustellen, dass die Konzepte nicht als evidenzbasiert gelten [3]. Sie erfüllen allerdings die Kriterien der *gesicherten arbeitswissenschaftlichen Erkenntnisse *für die Anwendung in der Praxis des Arbeitsschutzes, welche a) von der Zustimmung der Mehrheit der Fachexpert*innen auf dem Gebiet abhängen sowie b) von der Erprobung und Bewährung der Erkenntnisse in der betrieblichen Praxis, sofern sie zur Verbesserung des Gesundheitsschutzes beitragen [2, 10].

Als positive Wirkungen des Suchtpräventionsprogramms wurden z. B. in der Erhebung zur Expertise von den Betrieben genannt: das gestiegene Bewusstsein für Sucht- und Gesundheitsgefährdungen durch Alkohol, die Enttabuisierung von Suchtgefährdung und Alkoholabhängigkeit, das konsequente Anbieten von Hilfe, die Reduzierung des Suchtmittelkonsums und die Verbesserung des Führungsverhaltens [10]. Die Senkung von krankheitsbedingten Abwesenheitszeiten sowie Kosteneinsparungen werden an anderer Stelle betont [14].

Die betriebliche Suchtprävention ist der *effektivste Weg, erwachsene Menschen mit gezielten Präventionsanliegen* zu erreichen. Die *Qualifizierungen zum Umgang mit Alkoholproblemen* haben auf breiter Ebene Verständnis dafür geschaffen, dass die Alkoholkrankheit oder andere Formen der Abhängigkeit fachlich geeigneter Entwöhnungstherapien bedürfen [10]. Durch die konsequent mit Hilfeangeboten verbundenen Interventionen bei Auffälligkeiten am Arbeitsplatz wird alkohol- und suchtgefährdeten oder -kranken Beschäftigten frühzeitig eine Perspektive aufgezeigt, wie sie *mit betrieblicher Unterstützung eine Lösung ihrer Suchtprobleme in Angriff nehmen* können [5]. Auf diesem Wege verbessert sich die Prognose für den Erfolg von Entwöhnungstherapien insgesamt, vor allem aber von Angeboten im Bereich ambulanter und stationärer Kurzeittherapien [16].

In einer landesweiten Erhebung mit 2986 einbezogenen thüringischen Betrieben mit mehr als 50 Beschäftigten (Besch.) gaben 17 % der Mittelbetriebe mit weniger als 250 Besch., 38 % der größeren Mittelbetriebe mit bis zu 999 Besch. und 75 % der Großbetriebe mit über 1000 Besch. im Jahr 2019 an, ein Suchtpräventionsprogramm etabliert zu haben (*n* = 711; [15]). Vergleicht man die Ergebnisse mit der bundesweiten Erhebung zur Expertise aus dem Jahr 2003 [10], lässt sich ein deutlicher Anstieg vor allem in den Großbetrieben von seinerzeit lediglich 59 % auf 75 % feststellen.

## Daten zum riskanten Alkoholkonsum und Regulierungen im Betrieb

Für die Datenlage des riskanten Alkoholkonsums in Deutschland wurde auf quantitative Daten der Bundesgesundheitsberichterstattung und des epidemiologischen Suchtsurveys sowie auf quantitative wie qualitative Studien der Arbeits‑, Gesundheits- und Stressforschung zurückgegriffen. Analysen zu Gesundheitsressourcen, zur psychischen Belastung und Beanspruchung sowie zum Bewältigungsverhalten liefern wichtige Grundlagen für dieses Kapitel.

### Riskanter Alkoholkonsum – Ursachen in der Arbeitssituation

In einer Reihe von Studien zeigte sich *ein enger Zusammenhang zwischen Berufsarbeit, Arbeitsbedingungen und Suchtmittelgebrauch*, insbesondere zum Alkoholkonsum [11, 12, 17]. Dabei kann der Alkohol unterschiedlich funktionalisiert werden. Leicht verfügbar, wird er zur *Anregung* bei der Arbeit ebenso eingesetzt wie zur *Entspannung, zur Entlastung und vor allem auch zur Belohnung *nach einem stressigem Arbeitsalltag [18, 19]. Alkoholische Getränke haben den Effekt, dass eine schnelle gezielte Bedürfnisbefriedigung mit kurzfristig positiver Wirkung erreicht werden kann [20]. Das führt dazu, dass die Grenze des risikoarmen Konsums recht schnell überschritten wird und vermehrt riskante Mengen konsumiert werden [18]. Unter gesundheitlichen Aspekten ist gemäß der Definition des Robert Koch-Instituts (RKI) ebenso wie nach den S3-Leitlinien der Konsum als riskant zu betrachten, wenn der Schwellenwert von 12 g Reinalkohol für Frauen bzw. 24 g für Männer pro Tag überschritten wird [4, 21]. Oberhalb dieser Grenzen steigen auf Dauer die *gesundheitlichen Risiken *(z. B. das Krebsrisiko; [21]). Darüber hinaus gibt es gerade im Beruf *eine Reihe von Sicherheitsrisiken sowie soziale Risiken,* wie etwa Statusverluste oder durch Regelverstöße verursachte Sanktionen bis hin zum Arbeitsplatzverlust [9, 19].

Aussagen zum Umfang des riskanten Alkoholkonsums im Betrieb sind aufgrund der zur Verfügung stehenden uneinheitlichen Datengrundlagen schwer zu treffen. Nach Auswertungen der GEDA(Gesundheit in Deutschland aktuell)-Studie praktizieren 13,8 % der Frauen und 18,2 % der Männer mindestens einmal pro Woche riskanten Alkoholkonsum. Bei Betrachtung der unterschiedlichen Altersgruppen zeigt sich, dass die höchste Prävalenz bei den 45- bis 64-Jährigen liegt [4]. Nach dem Epidemiologischen Suchtsurvey 2018 weist die 30-Tage-Prävalenz – das heißt der riskante Alkoholkonsum innerhalb der zurückliegenden 30 Tage – von Personen im berufsfähigen Alter einen höheren Wert bei Frauen (19,7 %) als bei Männern (16,7 %) auf^2^ [22]. Auf einen höheren Anteil von Frauen an den Risikokonsumenten kommt auch eine betriebsbezogene Studie in der Befragung der Beschäftigten einer Klinik anhand der Kriterien des AUDIT(Alcohol Use Disorders Indentification Test)-Scores. 28,7 % der Frauen gegenüber 19 % der Männer (*N* = 217) zeigten riskante Alkoholkonsummuster [23].

Als konstant erweist sich gegenwärtig der Schichtgradient: Frauen und Männer aus der oberen Bildungsgruppe (mit Hochschulabschluss) weisen danach höhere Prävalenzen des riskanten Alkoholkonsums auf als jene aus unteren Bildungsgruppen^3^ [20, 23]. Die Tatsache, dass Rauschtrinken bei Frauen nicht mit dem sozioökonomischen Status ansteigt [22], lässt die Annahme zu, dass Frauen mit höherem Status ihren (riskanten) Alkoholkonsum eher rollenkonform z. B. mit einem „guten Glas Wein“ zelebrieren [18].

Beschäftigte, deren Arbeitsanforderungen mit Stressbelastungen und Fehlbeanspruchungen einhergehen, konsumieren Alkohol häufiger in riskantem Umfang. *Besonders belastend wirken sich anhaltender Zeit- und Leistungsdruck, inkonsistente Anforderungen und Rollenerwartungen sowie Gratifikationsdefizite aus* [12, 17]. Bei der Betrachtung des Gefährdungspotenzials einzelner Berufsgruppen und Belastungsbereiche zeigen sich die Probleme des riskanten Alkoholkonsums vor allem in spezifischen Dienstleistungsbereichen, z. B. bei Flugbegleiter*innen, Pflegepersonal und Servicekräften mit hohen emotionalen Belastungen [25, 26].

### Regulierung und Reduzierung des Alkoholkonsums am Arbeitsplatz

Führungskräfte haben die Pflicht, in ihrem Verantwortungsbereich Tätige über Arbeitssicherheit und Gesundheitsschutz bei der Arbeit zu unterweisen (§ 12 ArbSchG). Unter anderem darüber, dass sich Beschäftigte nach § 15 DGUV V1 (Deutsche gesetzliche Unfallversicherung Vorschrift 1) durch Alkohol, Drogen oder andere Rauschmittel nicht in einen Zustand versetzen dürfen, in dem sie sich oder andere gefährden könnten. Sie sind nach § 7 derselben Vorschrift vom Arbeitsplatz zu entfernen, wenn ein sicheres Arbeiten nicht mehr gewährleistet ist [27]. Fachliche Unterstützung für die Unterweisung können Führungskräfte von den betrieblichen Fachkräften im Arbeitsschutz oder der Beratungsstelle für Suchtfragen erhalten. Diese können *praxisbezogene Informationen zur Vorbeugung von Sucht- und Sicherheitsproblemen *einbringen [1, 9, 28].

Angesichts der steigenden technischen und mentalen Anforderungen wird die Arbeit unter Alkoholeinfluss in vielen Bereichen kritisch gesehen und deshalb reguliert. Generelle Verbote des Konsums von Alkohol sind im Arbeitsschutz nur bei speziellen Tätigkeiten vorgegeben. Betrieblich ausgesprochene Verbote müssen angemessen sein und unterliegen mit wenigen Ausnahmen der Mitbestimmung [29, 30]. In einer Reihe von Betrieben ist man bemüht, die Beschäftigten dafür zu gewinnen, aus Verantwortung Grenzen zu setzen und z. B. in spezifischen Situationen ganz auf den Konsum von Alkohol zu verzichten. Die Suchtprävention vermittelt dazu einfache selbstbestimmt einzuhaltende Regeln, wie z. B. die der *Punktnüchternheit: 0 Promille bei der Arbeit und auf den Arbeitswegen* [9].

## Interventionen bei Auffälligkeiten in Arbeitssituationen

### Frühzeitige Interventionen – Fürsorge‑, Klärungs- und Rückmeldegespräche

Betriebliche Interventionen setzen nach heutigen Standards nicht erst bei fortgeschrittener Alkoholgefährdung an. Veränderungen im Verhalten von Beschäftigten fallen im Arbeitsalltag in der Regel schon viel früher auf. Im Rahmen gesundheitsorientierter Führung können* (wiederholte) Auffälligkeiten im Arbeits- und Sozialverhalten von Beschäftigten *zum Anlass genommen werden, ein *fürsorgliches oder klärendes Gespräch *mit den Betroffenen zu führen (Tab. 1; [1, 9]). In dieser Phase können anhaltende Konzentrationsprobleme, nachlassende Zuverlässigkeit, wiederkehrende Unregelmäßigkeiten und Klagen darüber, dass alles immer schwieriger und stressiger wird, Anzeichen dafür sein, dass die betreffenden Personen sich schwertun, die anfallenden Aufgaben und Pflichten zuverlässig zu bewältigen. Hierfür können gemeinsam Lösungen gesucht werden. Wenn dazu weitergehender fachlicher Rat erforderlich ist, *übernimmt die Führungskraft eine Lotsenfunktion* zu den internen oder externen Unterstützungs- und Hilfeangeboten. Auch das ist Suchtprävention. Denn Untersuchungen zeigen, dass Beschäftigte bei psychischem Stress als Bewältigungshilfe und Selbstheilungsversuch häufiger zu Alkohol, Medikamenten oder anderen Suchtmitteln greifen [17, 18, 20]. *Um frühe Interventionszeitpunkte nutzen zu können, wurden die Stufenpläne durch Fürsorge- und Klärungsgespräche ergänzt *[1]. Auch *Kurzinterventionen zur Reduzierung des Alkoholkonsums *können hier bereits angebracht sein, bevor sich stärkere Störungen und Gesundheitsgefährdungen ausprägen [21, 31].

**Table 1:** 

Art der Intervention	Anlass	Inhalte und Ziele
**Bei akuter Beeinträchtigung der Sicherheit bei der Arbeit**		
Eingreifen der/des Vorgesetzten (verpflichtend), ggf. Entfernung vom Arbeitsplatz (DGUV V1 § 7 in Verbindung mit § 15)	Einsatzfähigkeit ist durch Suchtmittelkonsum oder Medikamentengebrauch eingeschränkt	Gefährdung abwenden
		Fürsorge
		Sicherheitsregeln beachten
	Arbeitssicherheit ist nicht gewährleistet, verbindliches Vorgehen erforderlich	Wiederholung vermeiden
		Anlassbezogene Unterweisung und/oder 1. Stufengespräch ansetzen
**Bei belastungs- und/oder gesundheitsbedingten Auffälligkeiten im Arbeits- und Leistungsverhalten**		
Fürsorgegespräch	Auffälligkeiten am Arbeitsplatz, z. B. in der Arbeitsbewältigung, im Arbeitsverhalten ohne (eindeutigen) Hinweis auf Risikokonsum/-verhalten	Fürsorge und Wertschätzung zum Ausdruck bringen
		Auffälligkeiten benennen
		Unterstützung anbieten
		Rückmeldegespräch ansetzen
Erstes Klärungsgespräch	Wiederholte Auffälligkeiten am Arbeitsplatz	Auffälligkeiten ernst nehmen
	Vernachlässigung von Arbeits- oder Dienstpflichten ohne (eindeutigen) Hinweis auf Risikokonsum/-verhalten	Fürsorge und Wertschätzung zum Ausdruck bringen
		Konkret Veränderungen einfordern/Absprachen
		Unterstützung anbieten
		Gesprächsnotiz aushändigen
		Rückmeldegespräch ansetzen
Weitere(s) Klärungsgespräch(e) (im erweiterten Kreis)	Fortgesetzte Auffälligkeiten, Vernachlässigung von Arbeits- oder Dienstpflichten ohne (eindeutigen) Hinweis auf Risikokonsum/-verhalten	*Entwicklung auswerten:* Auffälligkeiten gravierend u./o. unverändert
	Verstöße gegen Absprachen	Nächste Veränderungsschritte verabreden
		Verbindliches Unterstützungsangebot
		Folgegespräch vereinbaren
		Fachleute (aus dem Unterstützersystem) einbinden
Rückmeldegespräche	Bei positiver Entwicklung nach vorausgegangenen gesundheitsbezogenen Gesprächen	Auswertung der Entwicklung seit dem vorangegangenen Gespräch
		Ggf. weiteres Unterstützungsangebot
		Ggf. weitere Gespräche vereinbaren bzw. ankündigen
**Bei riskantem Konsum und Suchtgefährdung**		
1. Stufengespräch bei riskantem Suchtmittelgebrauch und Suchtgefährdung	Sucht(mittel)bedingte Auffälligkeiten am Arbeitsplatz	Fürsorge und Wertschätzung
	Anzeichen (möglicher) Suchtgefährdung	Auffälligkeiten benennen
		Suchtmittelgebrauch bzw. Suchtgefährdung ansprechen
		Unterstützung anbieten
		Anlassbezogene Unterweisung ansetzen und über Gefährdungen aufklären
		Rückmeldegespräch ansetzen
2. bis 4./5. Stufengespräch (im erweiterten Kreis)	Sucht(mittel)bedingte Auffälligkeiten am Arbeitsplatz	Auffälligkeiten in Verbindung mit riskantem Konsum/Verhalten benennen
	Anzeichen (möglicher) Suchtgefährdung	Suchtgefährdung aufzeigen
		Konkrete Veränderungsschritte vereinbaren
		Unterstützung anbieten
		Beratung und Therapie (dringend) empfehlen
		Ggf. Sanktionen bei Fehlverhalten ankündigen und konsequent umsetzen
		Rückmeldegespräche ansetzen
**Zur Wiedereingliederung während/nach krankheitsbedingter Abwesenheit**		
Wiedereingliederungsgespräch am Arbeitsplatz (im erweiterten Kreis)	Rückkehr nach *längerer *Abwesenheit durch Erkrankung und Therapie	Gut wieder am Arbeitsplatz und im Team ankommen
	Verbindliches Gespräch zur Wiederaufnahme der Arbeit	Unterstützung aufzeigen
		Betriebliches Eingliederungsmanagement (BEM) empfehlen
		Begleitung des Eingliederungsprozesses durch Berater*in/Ansprechperson anbieten
		Vereinbarungen treffen

Personalverantwortlichen und anderen Verfahrensbeteiligten werden* Leitfäden für die Interventionen und die Gesprächsführung* zur Verfügung gestellt, die* Handlungssicherheit *bieten [1]. Außerdem werden sie speziell geschult, um wirksam intervenieren zu können. Kompetenzen zur *lösungsorientierten bzw. motivationalen Gesprächsführung *werden gefördert [21, 32]. Die Qualifizierung der Führungskräfte ist ein kritischer Erfolgsfaktor für die Umsetzung des Suchtpräventionsprogramms. Je größer ihr Anteil ist, umso verbindlicher und wirksamer werden Präventionsangebote und Interventionen eingesetzt [5].

### Verstöße gegen die Arbeitssicherheit

Aktuelle Suchtvereinbarungen legen darüber hinaus den Verfahrensablauf bei Verstößen gegen die Arbeitssicherheit fest. Vorgesetzte müssen nach § 7 DGUV V1 eingreifen, *wenn Beschäftigte unter dem Einfluss von Alkohol oder anderen Rauschmitteln sich oder andere gefährden könnten *und ein sicheres Arbeiten nicht mehr gewährleistet ist (Tab. 1; [27]). Führungskräfte nutzen den dafür vorgesehenen Interventionsleitfaden, um sach- und regelgerecht vorzugehen, wenn sie z. B. in unklaren Situationen die Entscheidung treffen müssen, Beschäftigte, die unter Suchtmitteleinfluss stehen, vom Arbeitsplatz zu entfernen [1, 32]. Nach einem solchen Vorfall ist die Führungskraft gehalten, den Stufenplan mit einem Gespräch der 1. Stufe einzuleiten.

### Stufengespräche und Wiedereingliederung

Sobald Auffälligkeiten im Arbeits- und Leistungsverhalten* mit problematischem Suchtmittel- oder Medikamentengebrauch oder suchtgefährdetem Verhalten* einhergehen, wird der *Weg über die gestufte Gesprächsfolge (Stufenplan) *eingeschlagen (Tab. 1; [1]). Das 1. Stufengespräch wird mit der auffällig gewordenen Person von der zuständigen Führungskraft unter 4 Augen geführt. Zu einem frühen Zeitpunkt besteht für Betroffene die gute Chance, *das beanstandete Verhalten durch eigene Initiative und ohne weiteren „Gesichtsverlust“* im Betrieb zu korrigieren. Bei positiver Verhaltensänderung erfolgen kein Eintrag in die Personalakte und keine weiteren Sanktionen [32].

Als Führungskraft ist es ratsam, eine *anlassbezogene Unterweisung* durch eine Fachkraft oder die Beratung für Suchtfragen anzusetzen. Darin wird die betroffene Person zu den möglichen Gefährdungen durch ihr Verhalten sowie über die Möglichkeiten, diesen vorzubeugen, aufgeklärt. Für die Teilnahme an der Unterweisung kann die Führungskraft einen schriftlichen Beleg einfordern [9].

Wer nach dem ersten Stufengespräch das Risikoverhalten fortsetzt, lässt erkennen, dass bei erneuten Auffälligkeiten weitere verbindliche Stufengespräche mit Hilfeangeboten notwendig werden. Im fortgeschrittenen Verlauf des Stufenplans wird die Vernachlässigung arbeitsvertraglicher bzw. dienstlicher Pflichten auch sanktioniert [1].

Durch frühzeitige Gespräche und Interventionen mit verbindlichen Unterstützungsangeboten können weitere Gefährdungen, Störungen sowie Ausfälle durch Abhängigkeitserkrankungen häufig abgewendet werden. Auch Kündigungen lassen sich dadurch nachweislich reduzieren [5]. Dagegen führen ungelöste Suchtprobleme im beruflichen Umfeld oftmals zu Stress und Konflikten bis hin zu Motivationsverlusten im Arbeitsteam. Deshalb gibt es für das kollegiale Umfeld spezielle Handlungshilfen für ein offenes Gespräch mit der betroffenen Person. So können sie ebenfalls Wege zur Hilfe aufzeigen und Veränderung anregen, ehe Problemsituationen eskalieren [33].

## Informations‑, Beratungs- und Unterstützungsangebote im Betrieb

### Suchtberatung und nebenamtliche Ansprechpersonen

Weder von den beteiligten Führungskräften noch vom kollegialen Umfeld wird erwartet, dass sie gegenüber einer suchtgefährdeten oder abhängigen Person selbst in die Beratungsrolle gehen.* Zuständig für die Unterstützungsangebote bei Suchtproblemen ist die interne Beratungsstelle oder Ansprechperson für Suchtfragen*, sofern es eine solche gibt. Andernfalls kann mit externen Fach- und Beratungsstellen kooperiert werden. Die Einrichtungen übernehmen die Information und Aufklärung zu Suchtthemen. Sie erörtern mit den Personalverantwortlichen die Hilfeangebote und Schritte im Interventionsverfahren [1, 4]. Sie beraten die Beschäftigten bei deren eigenen Suchtproblemen und solchen in ihrem Umfeld. Auf ihren Wunsch hin begleiten sie betroffene Personen vor dem Beratungsprozess und im Verlauf von ambulanten sowie stationären Therapiephasen. Teilweise werden auch Angehörige beraten. Schließlich werden die Beschäftigten bei ihrer Wiedereingliederung am Arbeitsplatz und in der Stabilisierungsphase begleitet.

Alle Beschäftigten haben zudem das Recht auf eine betriebsärztliche Beratung. Bei Gefährdungen in der Arbeitssituation können sie von den Fachkräften im Arbeitsschutz Unterstützung erwarten. Krankenkassen, Berufsgenossenschaften bzw. Unfallversicherungen sowie externe Beratungs- und Therapieeinrichtungen bieten den Betrieben ebenfalls Information und Beratung zur Suchtprävention an [1, 19, 28].

### Internes und externes Unterstützungssystem

Die internen Beratungs- und Ansprechpersonen für Suchtfragen bilden gemeinsam mit Fachkräften aus dem Arbeits- und Gesundheitsschutz, dem Personalwesen sowie mit Mitgliedern der Interessenvertretungen das betriebliche Unterstützungssystem [1]. In der Umsetzung des betrieblichen Gesundheits- und Eingliederungsmanagements arbeiten sie in der Regel bereits zusammen, teilweise auch mit externen Institutionen. Ein gut funktionierendes Unterstützungssystem stellt eine wichtige Ressource dar. Es kann u. a. Beschäftigten mit gesundheitlichen oder sozialen Einschränkungen zeitnah helfen, sachgerechte Problemlösungen oder weitergehende Hilfe zu finden, und fördert mit seinen Angeboten die Gesundheitskompetenz in der Organisation [1, 5, 9].

## Förderung der Gesundheit und Erweiterung der Gesundheitsressourcen

Die betriebliche Suchtprävention ist heute eng mit den Zielen und Angeboten des Personal- und Gesundheitsmanagements verknüpft. Sie wirkt daran mit, die Gesundheitskompetenz der Beschäftigten sowie der betrieblichen Organisation zu erweitern und zu stärken [6].

*Auf der Verhaltensebene* werden die Beschäftigten angeregt, ihre Belastungserfahrungen aus der Arbeitssituation sowie ihre Bewältigungsstrategien bewusst zu reflektieren. Kommen riskante Konsummuster in Bezug auf Alkohol, Tabak, Medikamente oder andere Suchtmittel zutage, werden Beschäftigte angeregt, einzeln oder gemeinsam in Teams *alternative Strategien für die Belastungsbewältigung unter Verzicht auf den Suchtmittelgebrauch* zu entwickeln. Über Angebote der Gesundheitsförderung und der Personalentwicklung lassen sich im Betrieb gezielt individuelle und kollektive Ansätze wie Coachings und Workshops nutzen, um sensibler gegenüber riskantem Alkoholkonsum und Gefährdungen durch Suchtmittelgebrauch im Arbeitskontext zu werden [6, 18]. Aus betrieblichen Informationen und Veranstaltungen werden darüber hinaus auch Anregungen zu einem verantwortungsvollen Umgang mit Alkohol in der Freizeit ins persönliche Umfeld hineingetragen [34].

*Auf der Verhältnisebene* kann die betriebliche Suchtprävention unter Bezug auf § 17 ArbSchG Verbesserungen in der Arbeitssituation anregen und an der Weiterentwicklung gesundheitsförderlicher Strukturen und Arbeitsbedingungen mitwirken [9] u. a. durch:

- Anregung und Abstimmung einer Vereinbarung für einen *suchtmittelfreien Arbeitsplatz* bzw. für die* Punktnüchternheit bei der Arbeit*;
- Einführung transparenter Regelungen zur Einschränkung des Alkoholkonsums und Suchtmittel- bzw. Medikamentengebrauchs am Arbeitsplatz sowie Regeln zur Vermeidung von Sicherheits- und Gesundheitsgefährdungen;
- Veränderung suchtfördernder Arbeitsbedingungen, insbesondere Abbau von Stress und Angst auslösenden Situationen, aber auch fehlende Anerkennung und Unterstützung vor allem im Vorgesetzten-Mitarbeiter-Verhältnis;
- Ausbau von betrieblichen Angeboten zur Stärkung individueller und organisationaler Gesundheitsressourcen sowie zur Erweiterung der Gesundheitskompetenz, insbesondere der Personalverantwortlichen und Entscheidungsgremien;
- Anregung einer gesundheitsförderlichen Organisationsentwicklung und zur Gestaltung motivierender und gesund erhaltender Arbeitsbedingungen.

## Fazit

Die betriebliche Suchtprävention ist ein komplexes Aufgabenfeld. Ihr Konzept basiert auf wissenschaftlichen Erkenntnissen, aber vor allem auf praktischen Wirkungserfahrungen. Sie ist wenig erforscht, aber hochwirksam im Sinne der Aufklärung und Problembewältigung bei Suchtproblemen im Betrieb und darüber hinaus zur Gestaltung gesünderer Arbeitsbedingungen.

## Footnotes

1 Im medizinisch-therapeutischen Kontext folgt man der Empfehlung der Weltgesundheitsorganisation (WHO) und ersetzt den Begriff „Sucht“ weitgehend durch den Begriff „Abhängigkeit“. Es wird von Alkohol‑, Drogen- oder Medikamentenabhängigkeit oder Abhängigkeitserkrankungen gesprochen. In den übrigen gesellschaftlichen Bereichen wird der Begriff *Sucht –* sowie die damit verbundenen Wortkombinationen wie „Suchtberatungsstellen“ oder „Suchtmittel“ – eher synonym oder sogar bevorzugt angewandt. In diesem Beitrag werden die Begriffe so benutzt, wie es im betrieblichen Alltag üblich ist. Unschärfen zwischen Definition und Praxis werden dabei in Kauf genommen.

2 Die Auswertungen beziehen sich auf die Bundesländer Hessen, Berlin, Nordrhein-Westfalen, Sachsen, Thüringen: „Zielpersonen des ESA 2018 waren deutschsprachige und in Privathaushalten lebende Personen im Alter zwischen 18 und 64 Jahren in Hessen (Geburtsjahrgänge 1954 bis 2000) bzw. im Alter zwischen 15 und 64 Jahren in Berlin, Nordrhein-Westfalen, Sachsen und in Thüringen (Geburtsjahrgänge 1954 bis 2003)“ [17].

3 Der Begriff „Bildungsgruppe“ orientiert sich an dem soziologischen Verständnis zur Eingruppierung von sozioökonomischen Gegebenheiten und hier insbesondere am Merkmal der Bildungsabschlüsse [24].
